# Supplementary material for: Scalable anisotropic cooling aerogels by additive freeze-casting
Source: Nat Commun. 2022 Sep 22;13:5553. doi: 10.1038/s41467-022-33234-8 (PMC9499976; doi:10.1038/s41467-022-33234-8)
Supplement: Supplementary file 1 — Supplementary Information [file 41467_2022_33234_MOESM1_ESM.pdf]

Supplementary Information

Scalable Anisotropic Cooling Aerogels by Additive Freeze-Casting

Kit-Ying Chan,[1,2] Xi Shen,[1,2,*] Jie Yang,[1] Keng-Te Lin,[3] Harun Venkatesan,[1] Eunyoung Kim,[1] Heng Zhang,[1] Jeng-Hun Lee,[1] Jinhong Yu,[4] Jinglei Yang,[1] Jang-Kyo Kim[1,5,*]

[1] Department of Mechanical and Aerospace Engineering, The Hong Kong University of Science and Technology, Hong Kong

[2] Department of Aeronautical and Aviation Engineering, The Hong Kong Polytechnic University, Hong Kong

[3] Centre for Translational Atomaterials, Swinburne University of Technology, Hawthorn, Melbourne, VIC 3122, Australia

[4] Key Laboratory of Marine Materials and Related Technologies, Ningbo Institute of Materials Technology and Engineering, Chinese Academy of Sciences, Ningbo 315201, China

[5] School of Mechanical and Manufacturing Engineering, University of New South Wales, Sydney, NSW 2052, Australia

Corresponding authors: Xi Shen (xi.shen@polyu.edu.hk) and Jang-Kyo Kim (mejkkim@ust.hk)

Supplementary Note 1. Microstructures and physical properties of WPU and composite aerogels

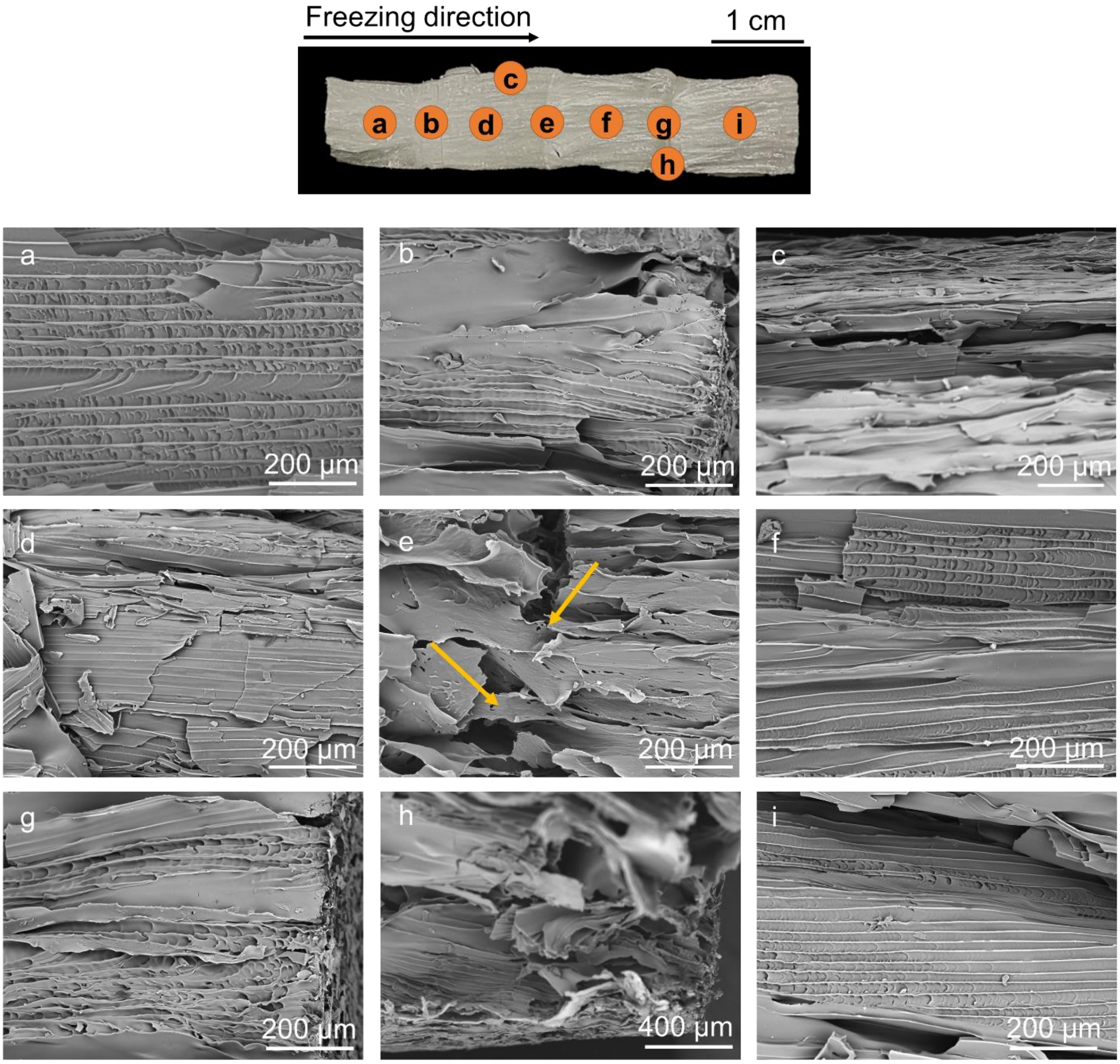

Supplementary Fig. 1. SEM images the pristine WPU aerogels fabricated using the additive freeze-casting technique with a moving cold source.

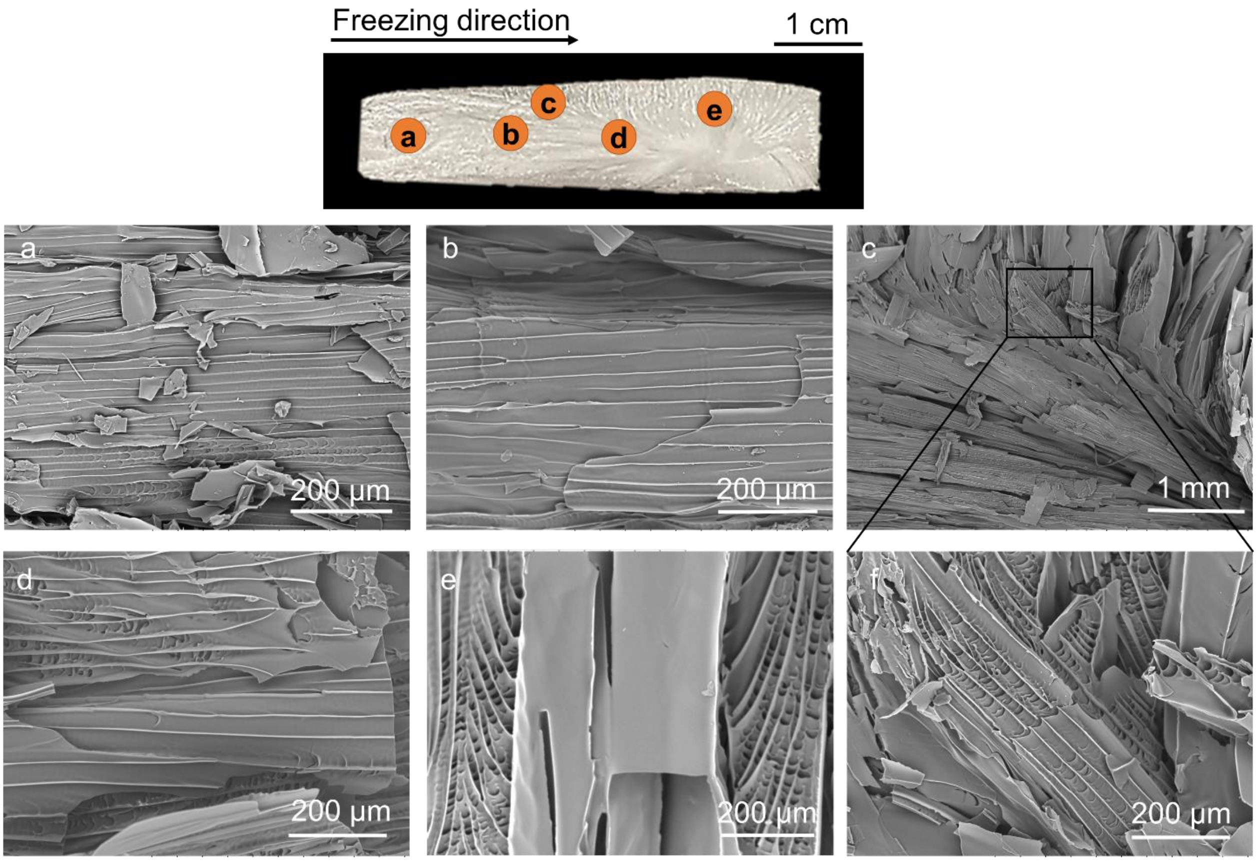

Supplementary Fig. 2. SEM images of the pristine WPU aerogels fabricated using the conventional unidirectional freeze-casting technique.

Although unidirectional freeze-casting has been widely used to produce aerogels having aligned pores, it lacks the ability to maintain consistent alignment and a uniform pore diameter along the long distance because of the short freezing distance. When the ice crystals grow away from the cold source, the temperature at the solidification front eventually becomes too high to drive the directional ice growth, resulting in randomly arranged pores at a distance far away from the cold source (Supplementary Figs. 2 c-e). Both our SEM observations and previous studies[1, 2] indicate that the critical freezing distance of each block required for uniform pore alignment should be ~15 mm or less.

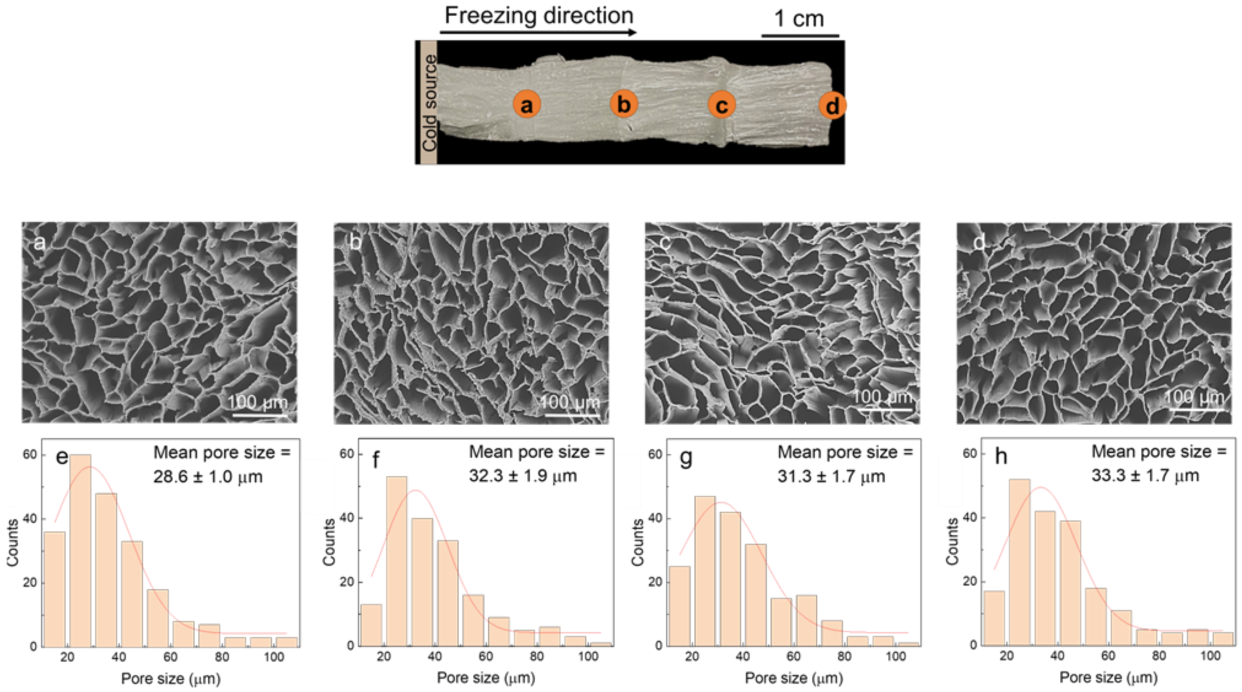

Supplementary Fig. 3. (a-d) Cross-sectional SEM images taken at different locations along the freezing direction of the WPU aerogel made by additive freeze-casting. (e-f) The corresponding pore size distributions measured from a-d, respectively, using Nano measurer software. The fitting with Guassian distribution indicates a consistent mean pore size of ~ 30 μm at different distances from the cold source.

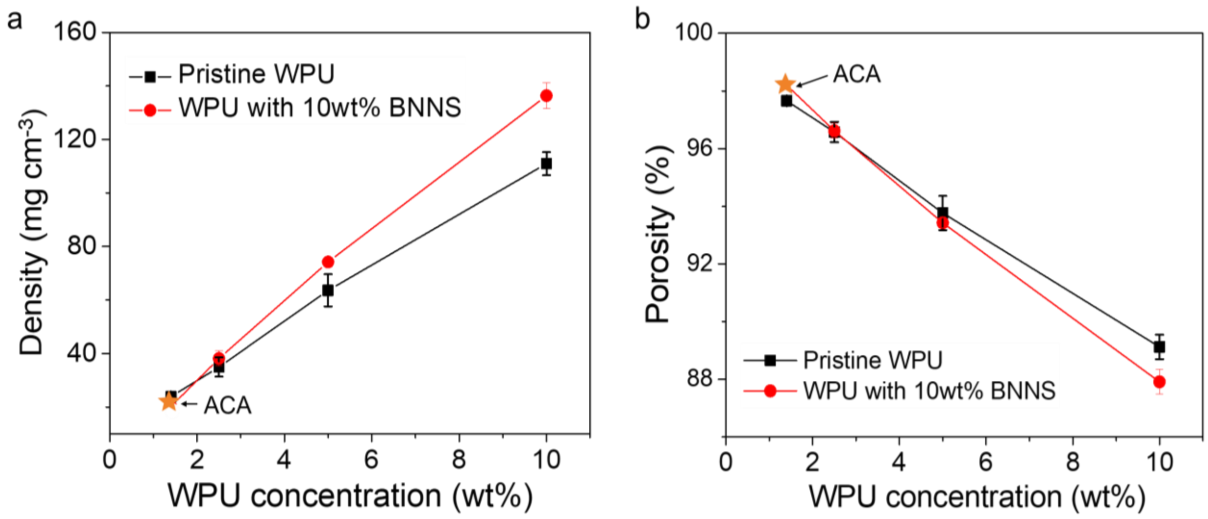

Supplementary Fig. 4. (a) Densities and (b) porosities of pristine WPU and composite aerogels with different compositions. Error bars represent standard deviations.

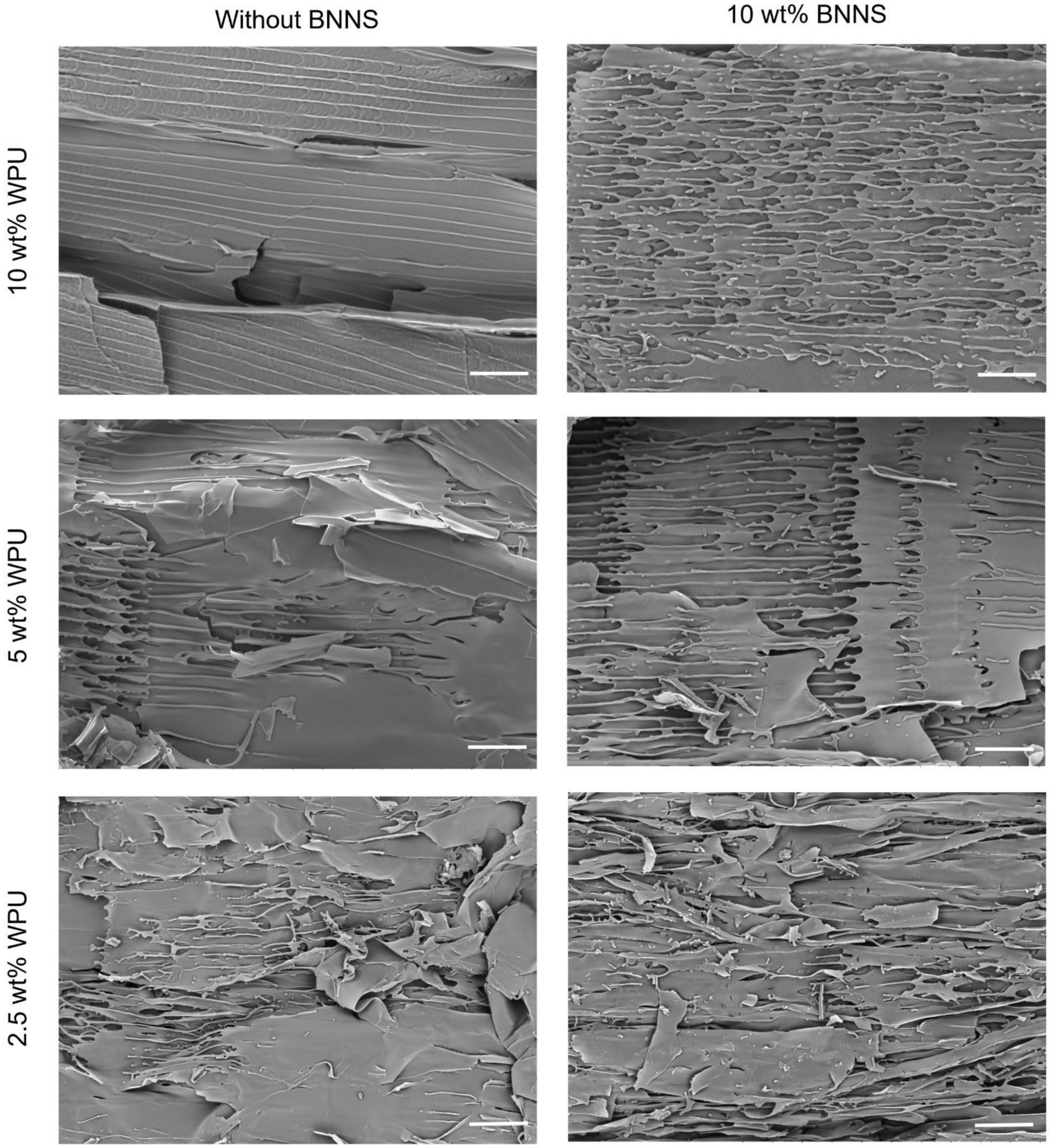

Supplementary Fig. 5. SEM images of pore channels of pristine WPU and composite aerogels with different compositions. Scale bars: 100 μm.

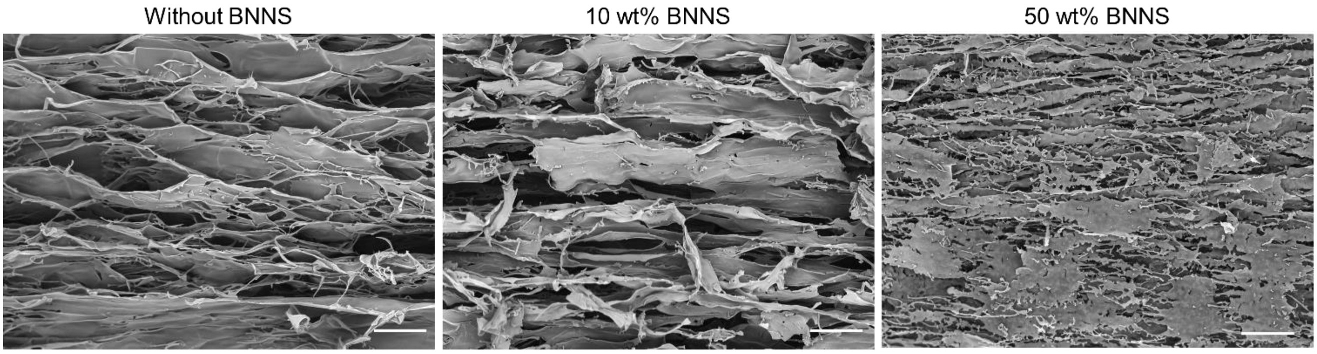

Supplementary Fig. 6. SEM images of pore channels of 1.4wt% WPU aerogels with different BNNS loadings. Scale bars: 100 µm.

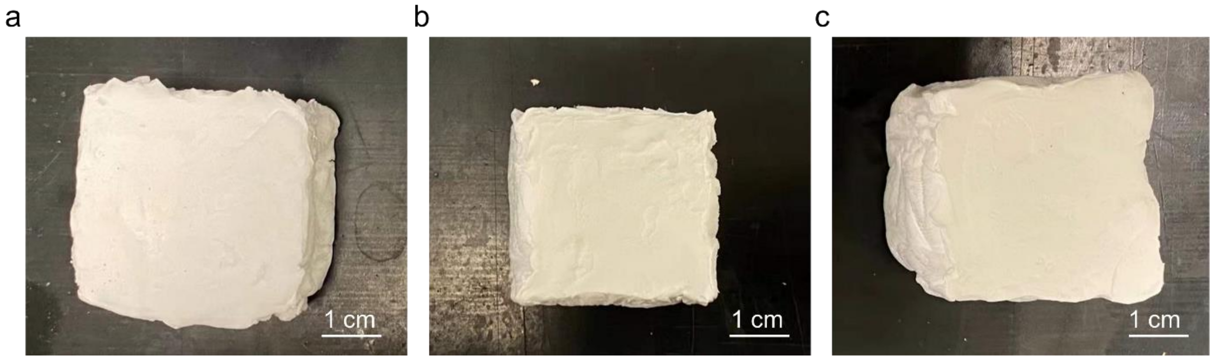

Supplementary Fig. 7. Digital photographs showing the shrinking of prinstine WPU and composite aerogels with different compositions: (a) 10 wt% WPU, (b) 1.4 wt% WPU and (c) 1.4 wt% WPU with 50 wt% BNNS loading. The volumetric shrinkage percentage of composite aerogels were calculated based on the sample volumes before and after freeze-drying.

Supplementary Note 2. Anisotropic thermal conductivities and compression performance of ACA

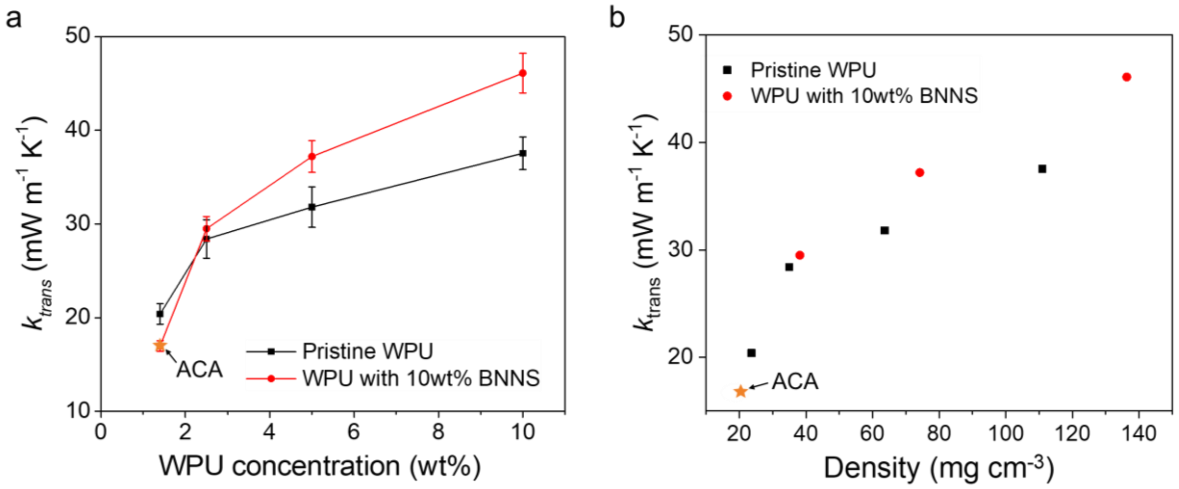

Supplementary Fig. 8. (a) Thermal conductivities of aerogels with different WPU concentrations. Error bars represent standard deviations. (b) Thermal conductivities of aerogels as a function of their densities.

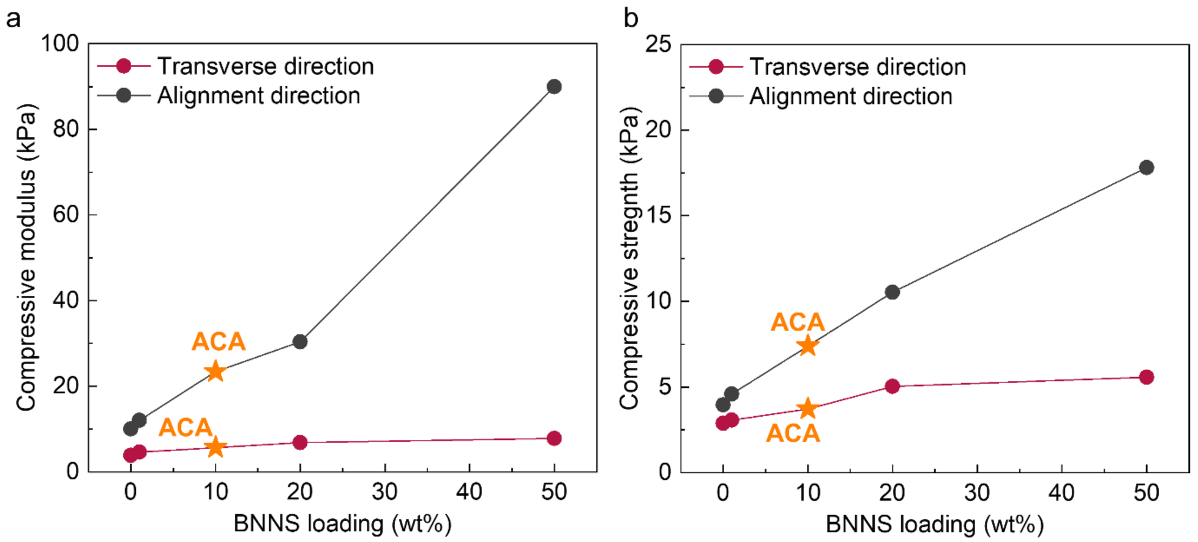

Supplementary Fig. 9. Compressive (a) moduli and (b) strengths at 50% of strains of aerogels with different BNNS loadings. The uniaxial compression tests were carried out on a universal testing machine (MTS Alliance RT-5) at a crosshead speed of 2 mm min^{-1} in accordance with ASTM − C165 – 07.

Supplementary Note 3. Solar reflectane of ACA

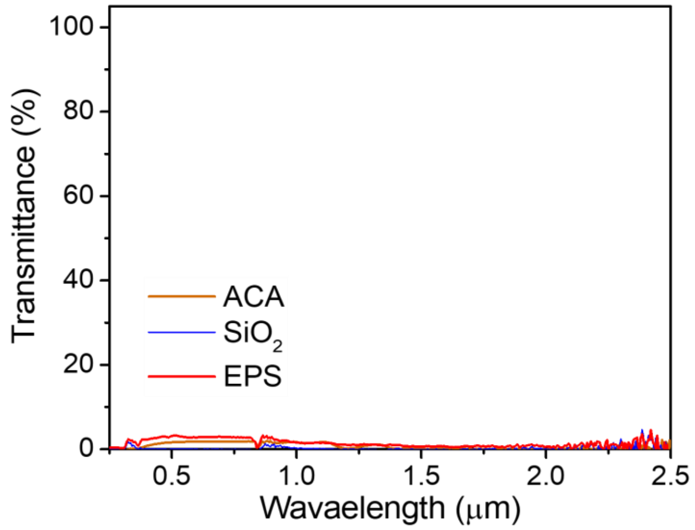

Supplementary Fig. 10. Solar transmittance spectra of ACA, commercial SiO_2 aerogel and EPS foam.

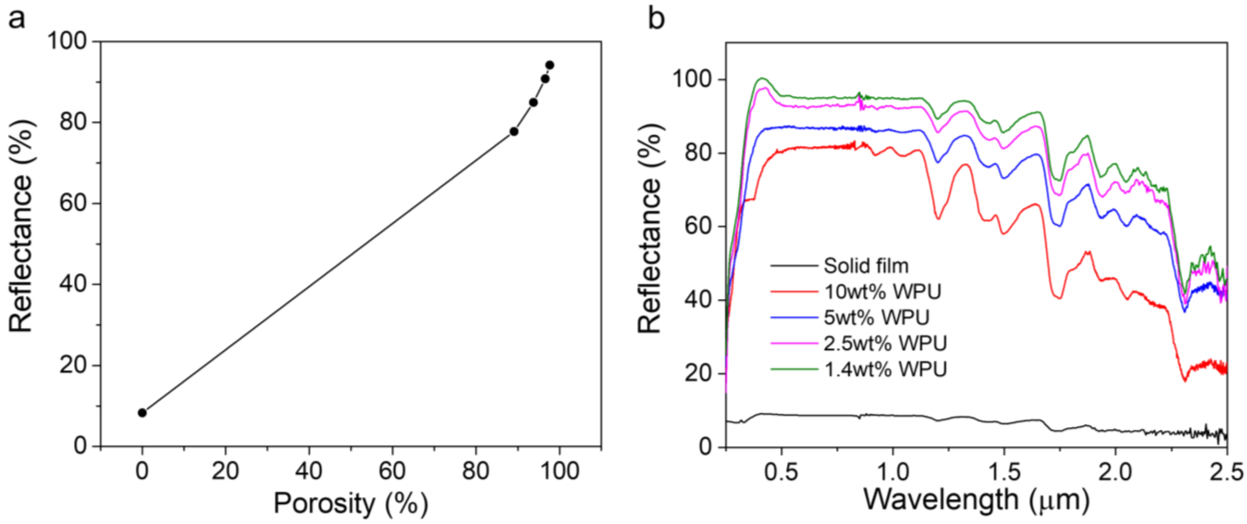

Supplementary Fig. 11. (a) Effect of porosity on reflectance of pristine WPU aerogels. (b) Solar reflection spectra of aerogels with different WPU concentrations. The porosities of solid film, 10 wt% WPU, 5 wt% WPU, 2.5 wt% WPU and 1.4 wt% WPU are 0, 89.1%, 93.8%, 96.2% and 97.7%, respectively.

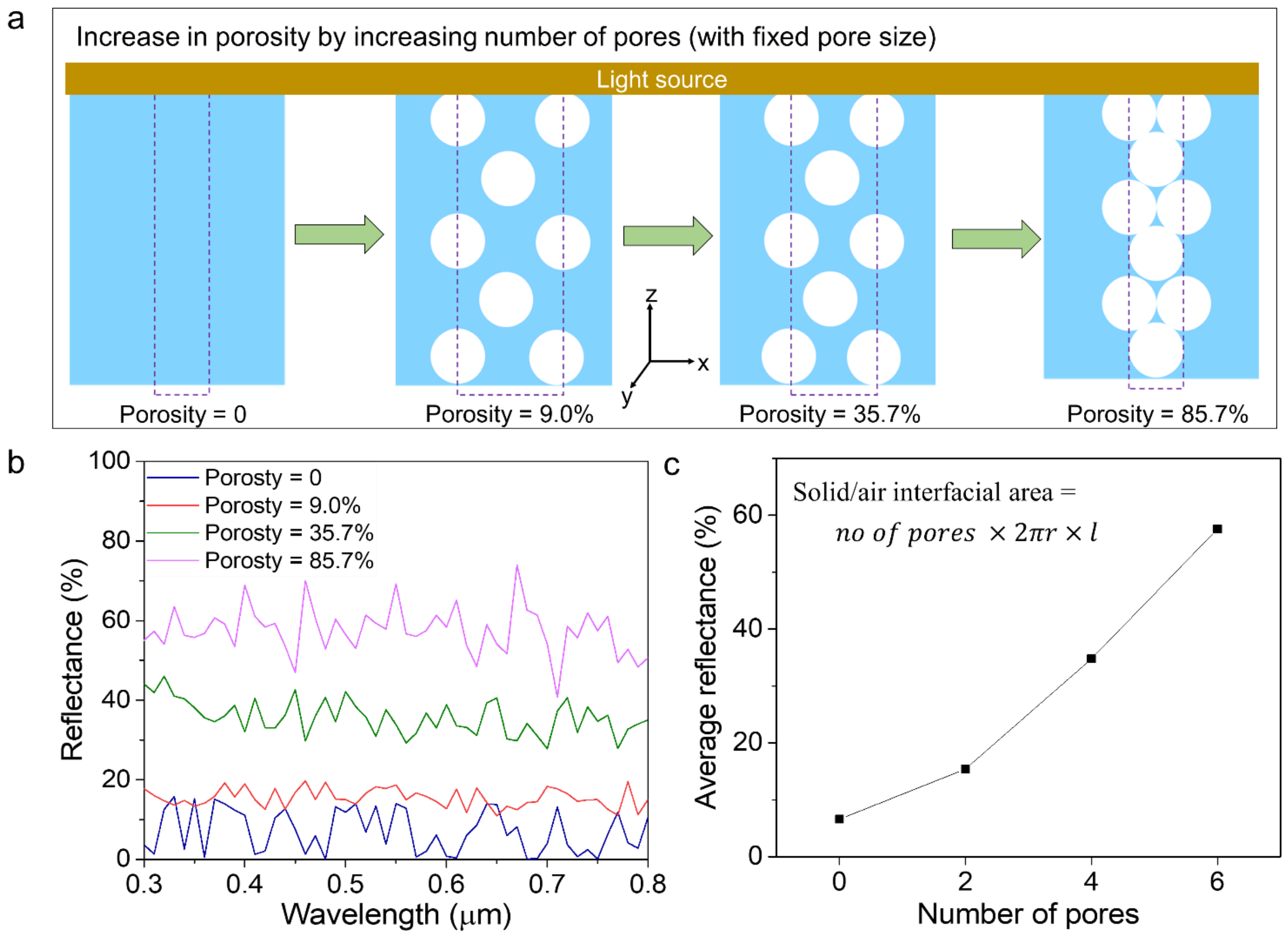

Supplementary Fig. 12. (a) Schematics of models used for finite-difference time-domain (FDTD) simulations. (b) Simulated reflection spectra of WPU aerogels having different porosities with the same pore size, as in (a). (c) Effect of number of pore on average reflectance of WPU aerogels. Since the pore diameter $(2r)$ and pore length (l) are the same in all models, the solid/air interfacial area is directly proportional to the number of pores.

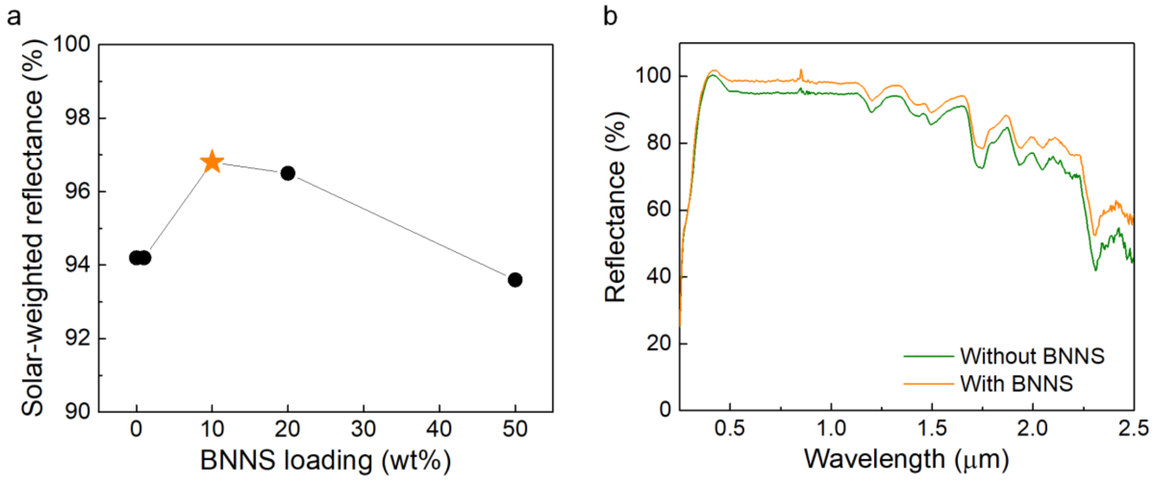

Supplementary Fig. 13. (a) Solar-weighted reflectance of 1.4 wt% WPU aerogels with different BNNS loadings. (b) Solar reflection spectra of 1.4 wt% WPU aerogels with and without BNNS.

Supplementary Note 4. Scalability analysis and materials cost estimation

The processing techniques involved in the synthesis of BNNS and additive freeze-casting, including liquid phase exfoliation, freeze-casting, and freeze-drying, are either industrially available techniques or easily scaled up for mass production.

First, the current work used the liquid phase exfoliation technique to attain BNNS from *h*-BN by ultrasonication, which is already well established for scalable production of BNNS in large quantities.[3] Second, the freeze-drying technique employed in this work to obtain aerogels is an industrially available technique widely used in pharmaceutical and food industries. Industry-scale freeze dryers having meter-scale chambers can accommodate large-scale products made from additive freeze-casting. Third, the additive freeze-casting technique established in this work can be scaled up for fabricating decimeter- or even meter-scale aerogels by increasing the lateral dimensions of the mold and the total freezing distance. The demonstration of large-scale fabrication of a decimeter-scale ACA panel is presented in Supplementary Fig. 14.

The whole process involved in additive freeze-casting is simple and does not require complicated equipment/apparatus or expensive solvents. Therefore, the cost for scaling up the freeze-casting set-up is considered low.[4] In addition, the raw materials used to fabricate ACA panels are available at cheap prices, as shown in Supplementary Table 1.

In view of the above analysis, the ACA developed in this work has a high potential for commercialization.

Supplementary Table 1. Cost estimation of raw materials for producing ACA panels.

**Raw material**	**Price**	**Amount per m^2**	**Price per m^2**
WPU resin	US$5.66 kg^{-1} (https://www.alibaba.com/product-detail/Waterborne-Polyurethane-Resin-Waterborne-Polyurethane-Hydroxyl_1600356457625.html?spm=a2700.galleryofferlist.normal_offer.d_title.7b913a5bBJ9gSJ&s=p)	0.18 kg	US$1.02
BNNS	US$50 kg^{-1} (https://www.alibaba.com/product-detail/Good-price-Boron-Nitride-Nanosheets-BN_1600430750000.html?spm=a2700.galleryofferlist.normal_offer.d_image.2d0c33adD6JZTv)	0.02 kg	US$1.00
		**Total:**	**US$2.02**

Note: The amounts per 1 m^2 of area are estimated based on the density of ACA (20.2 mg cm^{-3}), the composition of 90wt% WPU and 10wt% BNNS, and the thickness of 1 cm.

Supplementary Note 5. Decimeter-scale ACA panels for practical applications

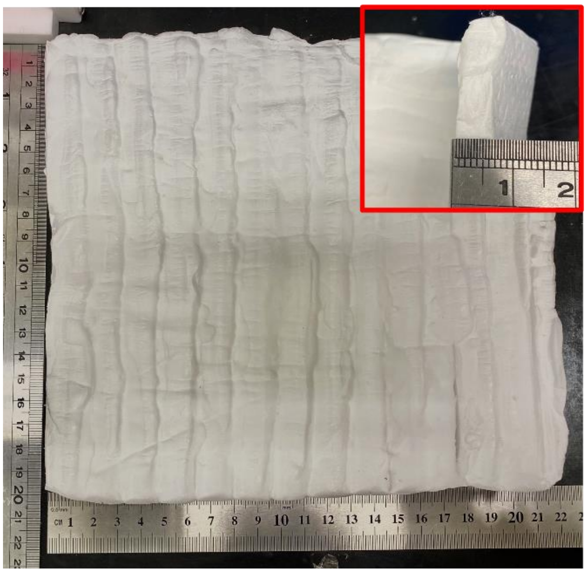

Supplementary Fig. 14. Digital photograph of the ACA panel fabricated by additive freeze casting. The laterial dimensions of the ACA panel are 20 cm × 20 cm with a thickness of 0.7 cm.

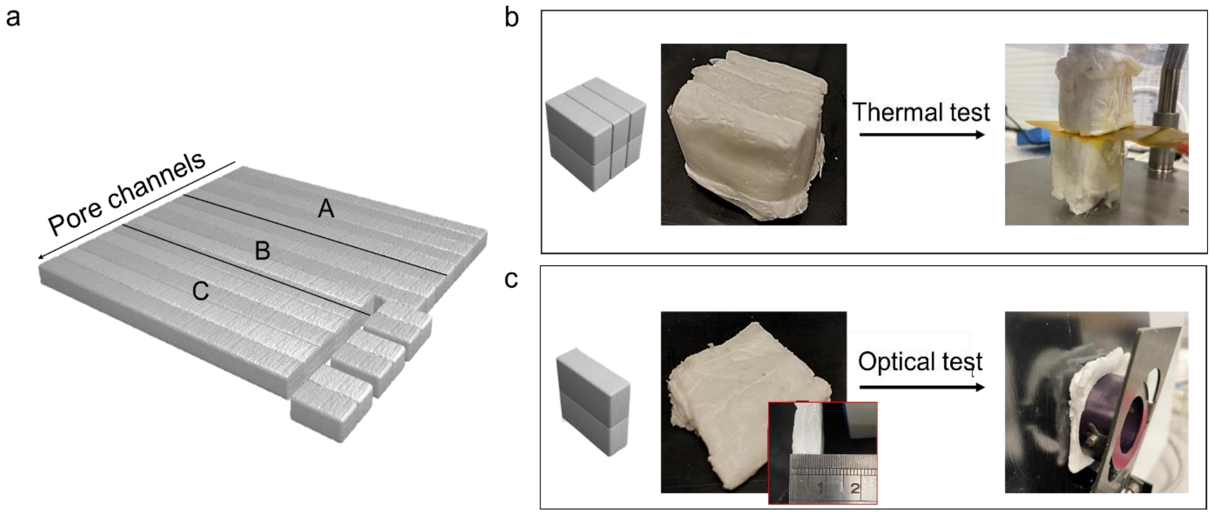

Supplementary Fig. 15. (a) Schematic showing the division of ACA panel. ACA samples for (b) thermal and (c) optical tests.

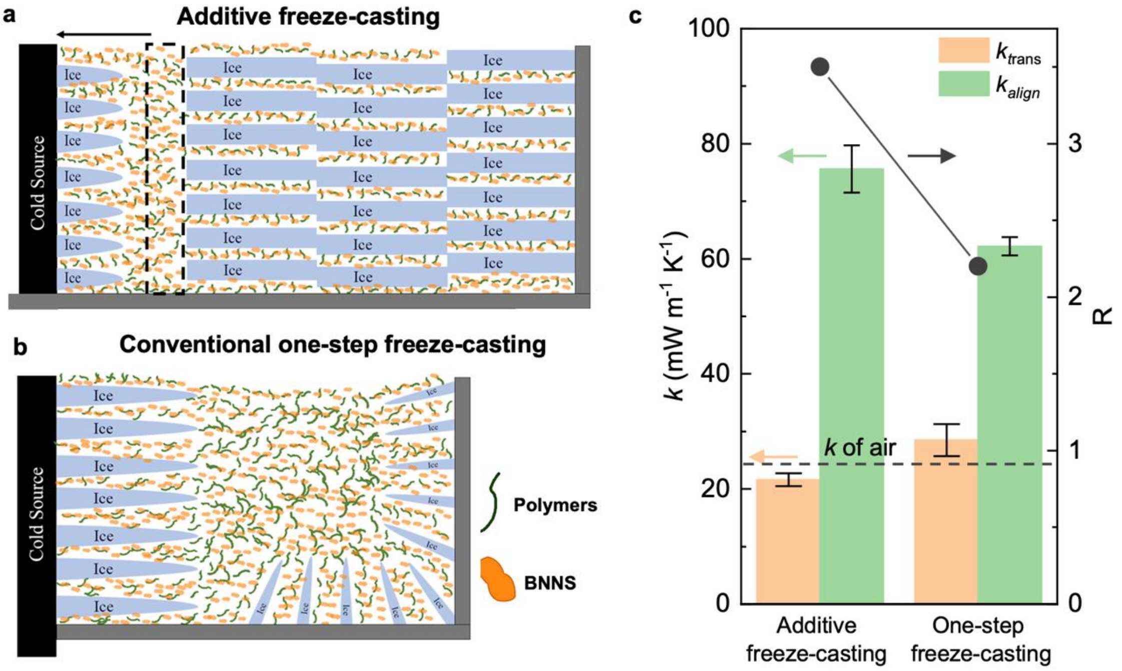

Supplementary Fig. 16. Schematics showing the ice crystal growths in (a) additive freeze-casting and (b) conventional one-step unidirectional freeze-casting. (c) Comparison of thermal conductivities and anisotropic factors of two aerogels made by different freeze-casting methods. Error bars represent standard deviations.

It should be noted that the complete freezing of decimeter-scale aerogels using one-step unidirectional freeze-casting could be extremely difficult, if not entirely impossible, because the temperature of solidification front tended to rise substantially once far away from the cold source. Multistep freeze-casting methods have been reported previously,[5-8] the stepwise ice growth was generated from a fixed cold source, limiting the overall length of the aligned pores to only a few centimeters.[5] To compare the thermal insulation performance, the aerogels were produced by both additive freeze-casting and conventional one-step freeze-casting with the same total freezing distance of 4.5 cm using moving and fixed cold sources, respectively.

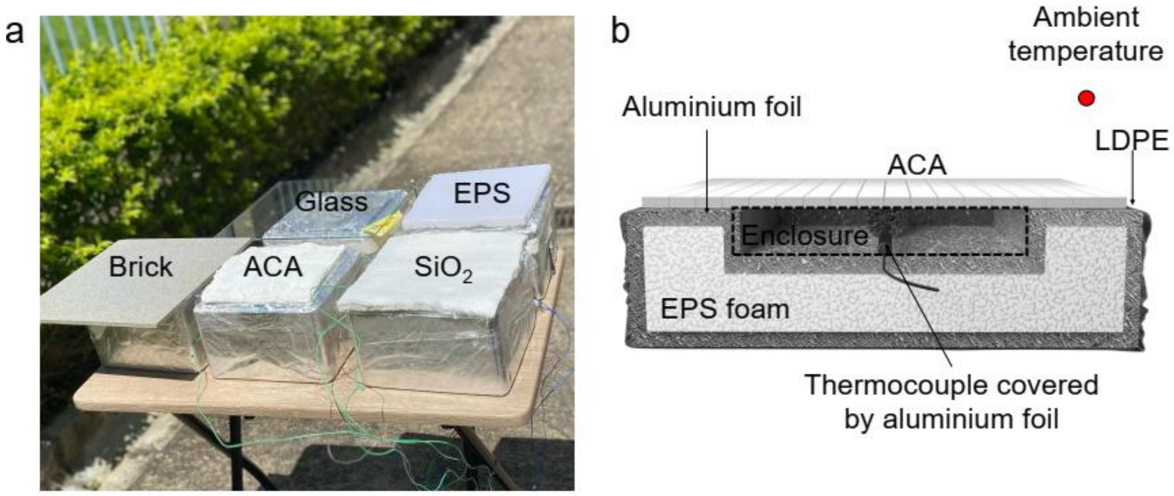

Supplementary Fig. 17. (a) Digital photograph and (b) schematic showing the set-up used for outdoor tests. All thermocouples were covered by aluminum foil to prevent exposure to sunlight.

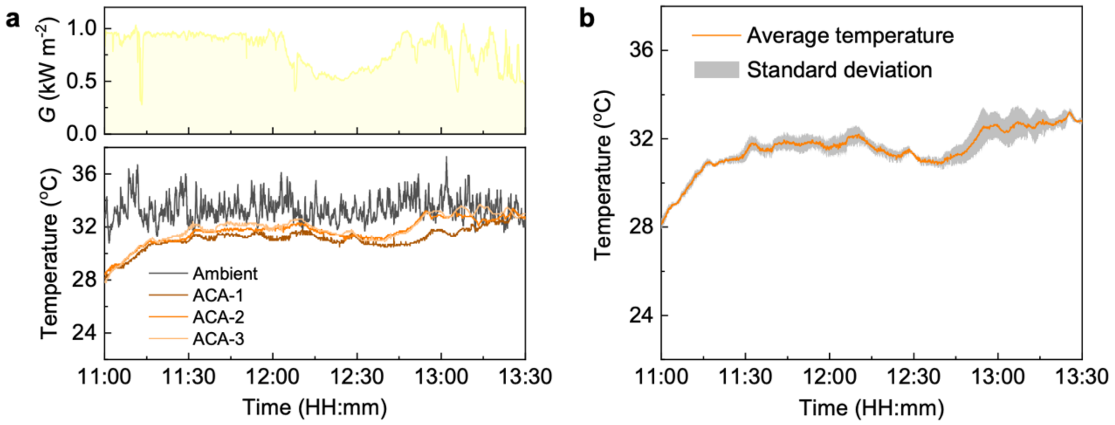

Supplementary Fig. 18. (a) Solar irradiance, *G*, and internal temperatures of three ACA samples measured in the outdoor tests. (b) Average temperatures of three samples and the standard deviations.

Supplementary Note 6. Cooling energy saving simulation

To evaluate the potential of ACA for cooling energy saving, a building energy simulation was performed using an open-source software EnergyPlus version 9.6.0. The building model without ACA (Supplementary Fig. 19) consisted of envelopes with baseline properties, including roofs (with an emissivity of 0.9, solar reflectivity of 0.3 and thermal conductivity of 0.16 W m^{-1} K^{-1}) and walls (with an emissivity of 0.9, solar reflectivity of 0.1 and thermal conductivity of 0.6 W m^{-1} K^{-1}) according to previous studies.[9, 10] For the building model with ACA, an ACA envelope with thermo-optical properties obtained in this work was placed on top of the baseline envelope. The indoor temperature was controlled at 26 °C by an HVAC system, which is an economic temperature recommended by the U.S. Department of Energy.[11] The cooling energy consumptions of the building models with and without ACA were calculated for different cities in China based on the climate data downloaded from the EnergyPlus website (https://energyplus.net/weather).

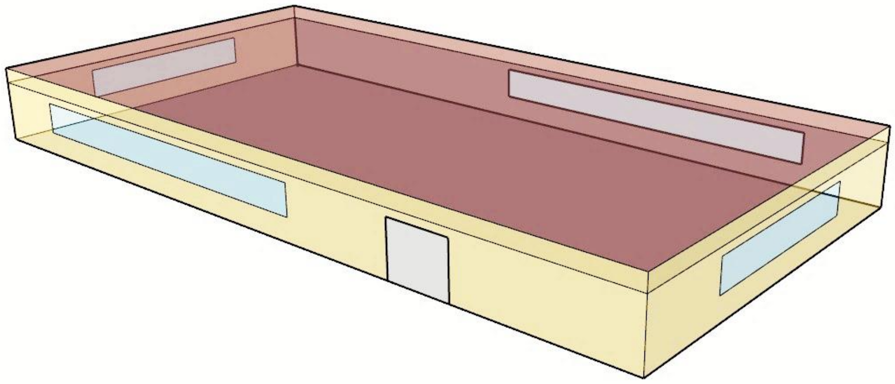

Supplementary Fig. 19. Schematic of a simplified residential house with floor area of 927 m^2 used for EnergyPlus simulation.

Supplementary Note 7. Weather resistance of ACA

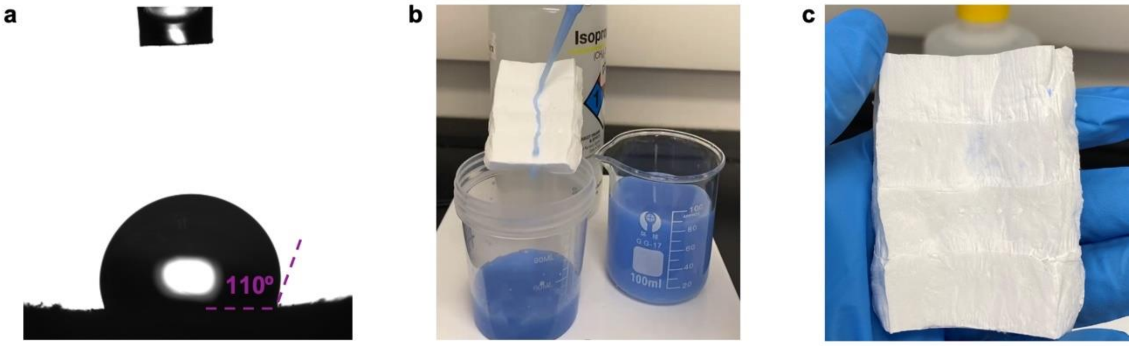

Supplementary Fig. 20. Water resistance of ACA. (a) Water contact angle of ACA. (b) Photograph showing a snapshot from the water resistance test of ACA. The whole test is shown in Supplementary Movie 3. (c) Photograph showing the surface of ACA after being washed with dyed water for 5 min. The surface of ACA remained intact with no noticeable changes, suggesting a good water resistance of ACA thanks to its hydrophobic surface.

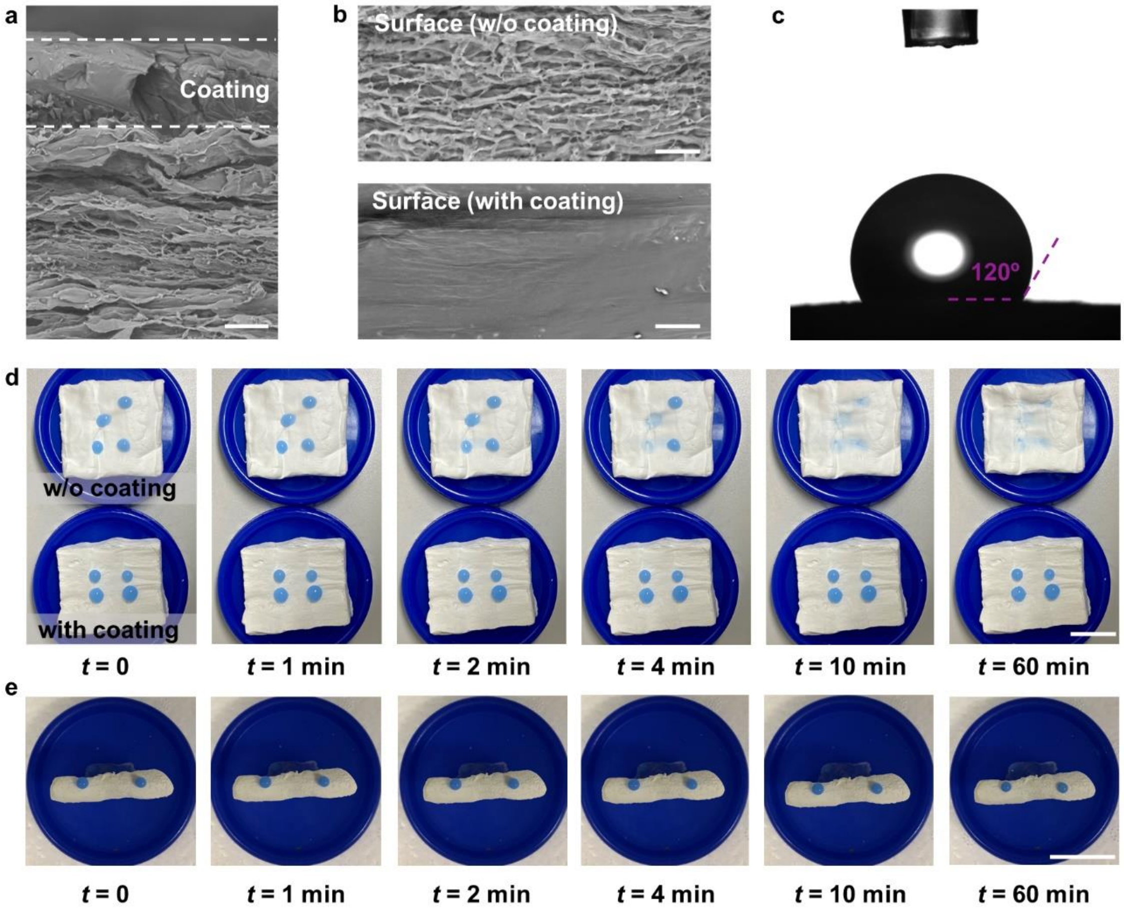

Supplementary Fig. 21. Water resistance of PU-coated ACA. (a) Cross-sectional SEM image of the PU-coated ACA (scale bar: 50 μm). The thickness of PU coating is ~ 80 μm. (b) Surface SEM images of PU-coated ACA compared to that without coating (scale bars: 20 μm). (c) Water contact angle of PU-coated ACA. (d) Photographs showing the changes of water droplets on the surfaces of ACA with and without PU coating with time. (e) Photographs showing the changes of water droplets on the PU-coated lateral surface of ACA. Scale bars in (d) and (e): 2 cm.

The ACA is compatible with commercial waterproofing coatings without losing the excellent thermal and optical properties. A transparent waterproofing coating (JY-S66, Shanghai Hanlong Company) for building envelopes was applied on the ACA surface via spray-coating. The PU coating had a thickness of ~ 80 μm (Supplementary Fig. 21a), blocking the surface pores of ACA (Supplementary Fig. 21b) to avoid water uptake through these pores. As shown in Supplementary Fig. 21c, the PU coating enhanced the water contact angle of ACA to 120°, making it highly water repellent (Supplementary Movie 4). Moreover, the water droplets applied on the coated surface remained unchanged after 60 minutes, demonstrating an excellent waterproof characteristic (Supplementary Fig. 21d). By contrast, the water droplets were gradually absorbed into the ACA without coating through its surface pores (Supplementary Fig. 21d).

Supplementary Table 2. Physical and thermal properties of ACA and PU-coated ACA. Errors represent standard deviations.

Sample	Density (mg cm^{-3})	Porosity (%)	Thermal conductivity (mW m^{-1} K^{-1})	
			Transverse	Alignment
ACA	20.2 ± 1.3	98.2 ± 0.1	17.0 ± 0.6	98.4 ± 7.2
PU-coated ACA	22.0 ± 1.0	98.0 ± 0.1	18.6 ± 0.7	101.3 ± 3.9

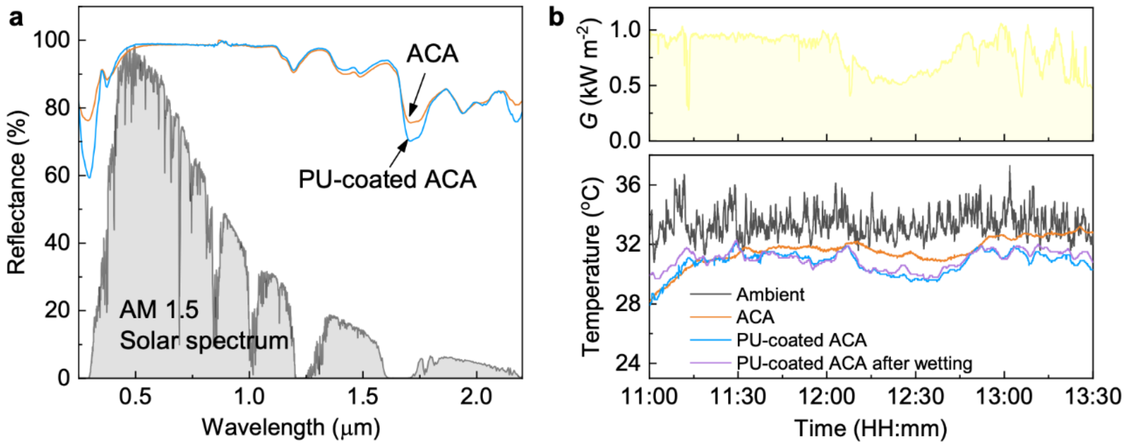

Supplementary Fig. 22. (a) Solar reflection spectra of ACA and PU-coated ACA. (b) Solar irradiance, *G*, and internal temperature changes in ACA, PU-coated ACA, and PU-coated ACA after wetting during the outdoor tests. For the last sample, the surface was continuously wetted by water droplets for 5 min as demonstrated in Supplementary Movie 4 prior to the outdoor test.

The waterproofing PU coating only marginally increased the thermal conductivity (Supplementary Table 2) while it had no adverse effect on the high solar reflectance (Supplementary Fig. 22a) of ACA thanks to its thin and transparent nature. Outdoor tests were also performed to compare the insulation performance of ACA and PU-coated ACA under practical conditions. The PU-coated ACA showed an internal temperature variation much the same as that without coating in the outdoor test even after wetting prior to the test (Supplementary Fig. 22b), showing a neglectable effect of PU coating on the practical insulation performance.

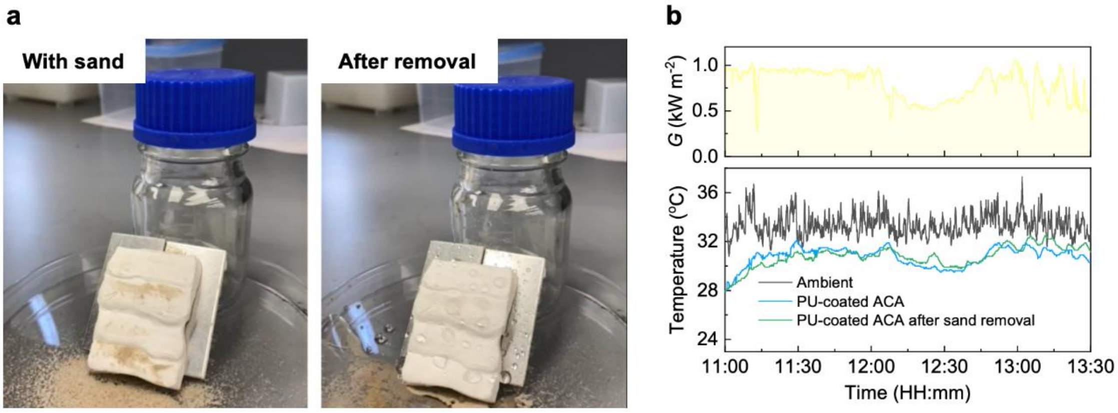

Supplementary Fig. 23. (a) Photographs showing the surface of ACA before and after removal of sand granules by physical cleaning with water. (b) Solar irradiance, *G*, and internal temperature changes in PU-coated ACA before and after sand removal in the outdoor tests.

The accumulation of dust could pose a challenge to the insulation performance of ACA as it reduces the solar reflectance performance. The common approach designed to address dust accumulation encompasses hydrophobic surfaces to allow easy removal of dust through physical cleaning.[12] The sand granules sprinkled on the PU-coated ACA surface to simulate accumulated dust were easily washed away by running water owing to its hydrophobic nature (Supplementary Fig. 23a). The undamaged surface obtained after sand removal gave rise to internal temperatures quite similar to those recorded before sand removal in the outdoor tests (Supplementary Fig. 23b), signifying the high durability of ACA panels for long-term usage under different weather conditions.

Supplementary Note 8. Comparison of overall performance of ACA with other materials

Supplementary Table 3. Thermal insulation and solar reflectance performance of ACA compared to other thermal insulation materials and high-reflectance or radiative cooling coatings.

Materials	Thermal conductivity (mW m^{-1} K^{-1})	Solar reflectance (%)	Contact angle
**Thermal insulation materials**			
**Cotton stalk fibers[13]**	58.5 – 81.5	/	/
**Durian peel and coconut coir[14]**	134.2	/	/
**Vermiculite, sunflower and wheat stalk[15]**	63 – 334	/	/
**Corn Stalk[16]**	51	/	/
**Wood waste[17]**	48 – 55	/	/
**PVA fiber aerogel[18]**	319.7	/	/
**Coal fly ash composite foam[19]**	51.1	/	/
**Polyurethane foam[20]**	20 – 30	/	/
**Expanded polystyrene foam[20]**	30 – 40	/	/
**Extruded polystyrene foam[21]**	25 – 35	/	/
**Glass wool[21]**	30 – 46	/	/
**Rock wool[21]**	33 – 46	/	/
**Liquid-crystalline nanocellulose aerogel[22]**	18	/	/
**GO/nanocellulose foam[23]**	15 (transverse)<br>170 (alignment)	/	/
**CNF/ZrP/RGO aerogel[24]**	18 (transverse)<br>45 (alignment)	/	/
**PVA/CNF aerogel[25]**	38.0	/	/
**CNF/MoS2 aerogel[26]**	28.09	/	Hydrophilic
**Attapulgite/gelatin composite aerogel[27]**	34.28 – 35.29	/	/
**ZIFs/PVA aerogel[28]**	32.7 – 36.3	/	/
**HAP/PVA aerogel[29]**	33.6 – 38.7	/	150°
**CNF aerogel[30]**	25.5	/	/
**SBC/CNF aerogel[31]**	28	/	/
**PI-BNC aerogel[32]**	23 (transverse)<br>44 (alignment)	/	/
**BCF-CNF aerogel[33]**	23	/	/
**Cellulose nanofibril/emulsion aerogel[34]**	15.5	/	/
**Cotton NFCs[35]**	39.6 – 45.5	/	/
**SiO_2-CNFs[36]**	13.8 – 20.1	/	Hydrophobic
**Silica granular aerogels[37]**	24	/	/
**BNNS/PVA aerogels[38]**	23.5	93.8	/
**Hypocrystalline ceramic aerogels[39]**	26	/	/
**PE aerogel[40]**	28	92.2	/
**PDMS/PE aerogel[41]**	32	96	155°
**Superhydrophobic cellulose aerogel[42]**	28	93	152°
**CNC aerogels[10]**	26	96	138°
**Hollow microfibers cooler[9]**	14	94	Hydrophobic
**Graphene-based foam[43]**	5.75	/	/

**Bioinspired polymeric woods[4]**	20.8	/	120° – 150°
**Nanowood[44]**	30 (transverse)<br>60 (alignment)	95<br>(0.4 – 1.1 μm)	/
**High-reflectance/radiative cooling coatings and films**			
**Silica aerogel/polyurethane film[45]**	/	69 – 89	101° – 135°
**Nanoporous polymer film[46]**	/	96.2	/
**Porous P(VdF-HFP)HP coatings[47]**	/	96	110°
**$BaSO_4$ nanocomposite paints[48]**	/	98.1	Water resistant
**TiO_2/Silica aerogel nanocomposite paint[49]**	29	90	142°
**ePTFE film/Ag layer[50]**	/	98	142°
**PMMA film[51]**	/	95	156°
**Electro-spun PAN nanofibers film[52]**	/	95	/
**Nanoporous composite fabric (NCF)[53]**	/	95	109° after 20 min
**SiO_2/EPDM porous composite film[54]**	/	96	162°
**TiO_2-free coatings[55]**	/	96	138.9°
**Superhydrophobic PTFE coating[56]**	/	93	165°
**TiO_2/glass microsphere/polymer emulsion[57]**	/	93.4	109.4°
**ACA (This work)**	16.9 (transverse)<br>98.4 (alignment)	97	110°

Supplementary Method. Synthesis of BNNS and characterization

The BNNS was synthesized by liquid phase exfoliation from bulk *h*-BN.[3, 58] In brief, 80 g *h*-BN powders were dispersed in 4 L deionized water, which was ultrasonicated using a sonicator (QSonica, Q500, 500 W, 20 kHz) for 120 minutes. The power applied to the suspension was controlled with a pulse mode at a 2 seconds on-time and 1 second off-time per pulse cycle. During the high intensity sonication process, the bulk *h*-BN powders were successfully exfoliated to few-layered BNNS. After ultrasonication, the exfoliated BNNS was separated from the unexfoliated *h*-BN powders using a centrifuge at ~2350 × g for 30 minutes. The supernatant was collected and dried to obtain BNNS, while the precipitate was subjected to the above sonication and centrifugation procedures until the complete exfoliation of *h*-BN powders.

The thickness and lateral size of BNNS were measured to be approximately 4 nm and in a range of 500 nm – 2 µm respectively, according to the atomic force microscopy (AFM) (Supplementary Figs. 24a,b). The morphology of well-exfoliated BNNS was examined using transmission electron microscopy (TEM) (Supplementary Fig. 24c). The hexagonal crystal structure of BNNS was indicated by the typical six-fold symmetry in the selected area electron diffraction (SAED) pattern. The abovementioned lateral size was confirmed by the SEM image (Supplementary Fig. 24d). The wide scan XPS survey spectrum (Supplementary Fig. 25a) showed peaks at 189, 396 and 531 eV corresponding to B1*s*, N1*s* and O1*s*, respectively. The binding energy was calibrated with the reference C1*s* peak at 284.5 eV. The high resolution B1*s* spectrum (Supplementary Fig. 25b) identified two major peaks of B-N and B-O bonds at 190.5 eV and 191.2 eV, respectively, and the B-N peak was also identified at 400 eV in the high resolution N1*s* spectrum (Supplementary Fig. 25c). The presence of B-O bonds might be due to the hydrolysis at boron edges to form pendant hydroxyl groups during exfoliation.[58-60] The Fourier-transform infrared spectroscopy (FTIR) was used to analyse the chemical bonds of BNNS (Supplementary Fig. 25d), showing strong absorption peaks at 1375 cm^{-1} and 810 cm^{-1} corresponding to the out-of-plane B−N bending vibrations and in-plane B−N−B stretching vibrations, respectively.[61, 62]

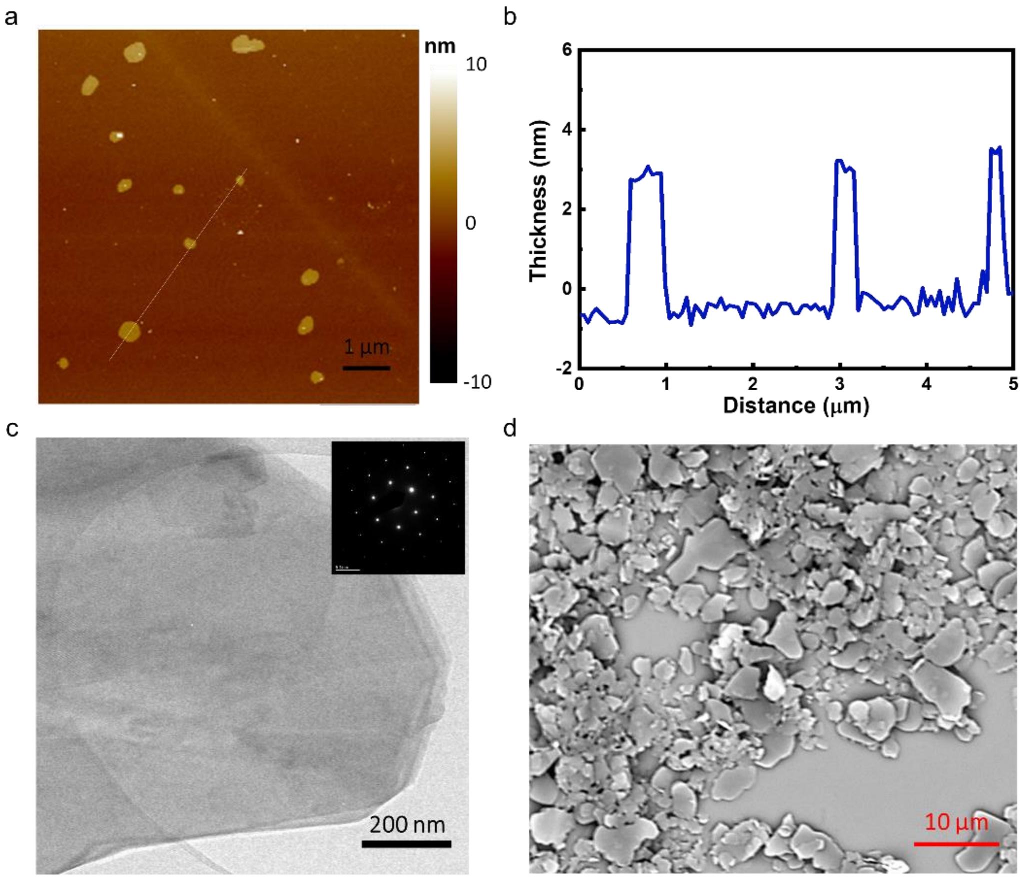

Supplementary Fig. 24. (a,b) AFM, (c) TEM with an SAED pattern in inset and (d) SEM images of as-prepared BNNS.

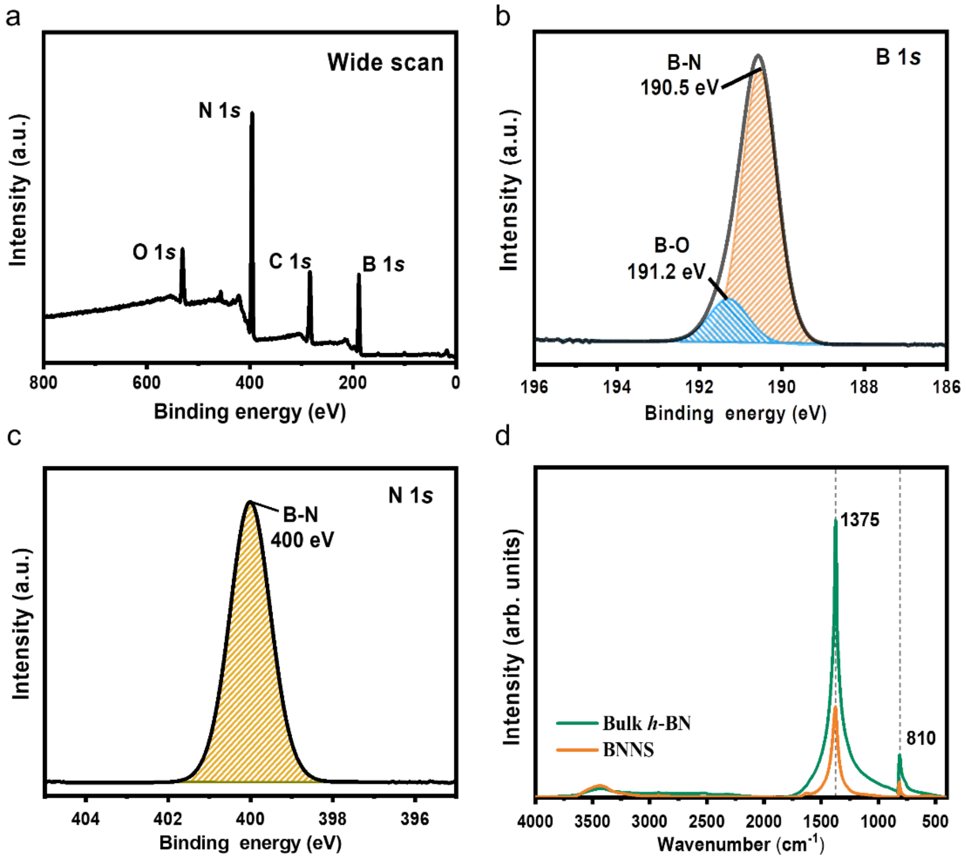

Supplementary Fig. 25. (a) General spectrum, high resolution (b) B1*s* and (c) N1*s* XPS spectra of as-prepared BNNS. (d) FTIR spectra of bulk *h*-BN and BNNS.

Supplementary References

1. Waschkies T, Oberacker R, Hoffmann MJ. Control of lamellae spacing during freeze casting of ceramics using double‐side cooling as a novel processing route. *J Am Ceram Soc* **92**, S79-S84 (2009).

2. Su FY, Mok JR, McKittrick J. Radial-concentric freeze casting inspired by porcupine fish spines. *Ceram* **2**, 161-179 (2019).

3. Coleman JN, *et al.* Two-dimensional nanosheets produced by liquid exfoliation of layered materials. *Science* **331**, 568-571 (2011).

4. Yu Z-L, *et al.* Bioinspired polymeric woods. *Sci Adv* **4**, eaat7223 (2018).

5. Christiansen CD, Nielsen KK, Bjørk R. Functionally graded multi-material freeze-cast structures with continuous microchannels. *J Eur Ceram Soc* **40**, 1398-1406 (2020).

6. Christiansen CD, Nielsen KK, Bjørk R. Novel freeze-casting device with high precision thermoelectric temperature control for dynamic freezing conditions. *Rev Sci Instrum* **91**, 033904 (2020).

7. Tang Y, Zhao K, Hu L, Wu Z. Two-step freeze casting fabrication of hydroxyapatite porous scaffolds with bionic bone graded structure. *Ceram Int* **39**, 9703-9707 (2013).

8. Ouyang A, *et al.* Highly porous core–shell structured graphene-chitosan beads. *ACS Appl Mater Interfaces* **7**, 14439-14445 (2015).

9. Zhong H, *et al.* Hierarchically Hollow Microfibers as a Scalable and Effective Thermal Insulating Cooler for Buildings. *ACS Nano*, **15**, 10076-10083 (2021).

10. Cai C, *et al.* Dynamically Tunable All-Weather Daytime Cellulose Aerogel Radiative Supercooler for Energy-Saving Building. *Nano Lett* **22**, 4106-4114 (2022).

11. Lin C, *et al.* All-weather thermochromic windows for synchronous solar and thermal radiation regulation. *Sci Adv* **8**, eabn7359 (2022).

12. Mandal J, Yang Y, Yu N, Raman AP. Paints as a scalable and effective radiative cooling technology for buildings. *Joule* **4**, 1350-1356 (2020).

13. Zhou X-y, Zheng F, Li H-g, Lu C-l. An environment-friendly thermal insulation material from cotton stalk fibers. *Energy Build* **42**, 1070-1074 (2010).

14. Khedari J, Nankongnab N, Hirunlabh J, Teekasap S. New low-cost insulation particleboards

from mixture of durian peel and coconut coir. *Build Environ* **39**, 59-65 (2004).

15. Binici H, Aksogan O, Dıncer A, Luga E, Eken M, Isikaltun O. The possibility of vermiculite, sunflower stalk and wheat stalk using for thermal insulation material production. *Therm Sci Eng Prog* **18**, 100567 (2020).

16. Ahmad MR, Chen B, Oderji SY, Mohsan M. Development of a new bio-composite for building insulation and structural purpose using corn stalk and magnesium phosphate cement. *Energy Build* **173**, 719-733 (2018).

17. Cetiner I, Shea AD. Wood waste as an alternative thermal insulation for buildings. *Energy Build* **168**, 374-384 (2018).

18. Hanif A, Diao S, Lu Z, Fan T, Li Z. Green lightweight cementitious composite incorporating aerogels and fly ash cenospheres–Mechanical and thermal insulating properties. *Constr Build Mater* **116**, 422-430 (2016).

19. Zhang R, Feng J, Cheng X, Gong L, Li Y, Zhang H. Porous thermal insulation materials derived from fly ash using a foaming and slip casting method. *Energy Build* **81**, 262-267 (2014).

20. Jelle BP. Traditional, state-of-the-art and future thermal building insulation materials and solutions–Properties, requirements and possibilities. *Energy Build* **43**, 2549-2563 (2011).

21. Villasmil W, Fischer LJ, Worlitschek J. A review and evaluation of thermal insulation materials and methods for thermal energy storage systems. *Renew Sust Energ Rev* **103**, 71-84 (2019).

22. Kobayashi Y, Saito T, Isogai A. Aerogels with 3D ordered nanofiber skeletons of liquid-crystalline nanocellulose derivatives as tough and transparent insulators. *Angew Chem Int Ed* **126**, 10562-10565 (2014).

23. Wicklein B*, et al.* Thermally insulating and fire-retardant lightweight anisotropic foams based on nanocellulose and graphene oxide. *Nat Nanotechnol* **10**, 277-283 (2015).

24. Wang D, *et al.* Biomimetic structural cellulose nanofiber aerogels with exceptional mechanical, flame-retardant and thermal-insulating properties. *Chem Eng J* **389**, 124449 (2020).

25. Huang Y*, et al.* Flame-retardant polyvinyl alcohol/cellulose nanofibers hybrid carbon aerogel by freeze drying with ultra-low phosphorus. *Appl Surf Sci* **497**, 143775 (2019).

26. Yang L*, et al.* Ultralight, highly thermally insulating and fire resistant aerogel by encapsulating cellulose nanofibers with two-dimensional MoS 2. *Nanoscale* **9**, 11452-11462 (2017).

27. Zhu J, *et al.* Thermal insulation and flame retardancy of attapulgite reinforced gelatin-based composite aerogel with enhanced strength properties. *Compos Part A Appl Sci* **138**, 106040 (2020).

28. Nabipour H, Nie S, Wang X, Song L, Hu Y. Zeolitic imidazolate framework-8/polyvinyl alcohol hybrid aerogels with excellent flame retardancy. *Compos Part A Appl Sci* **129**, 105720 (2020).

29. Guo W, Liu J, Zhang P, Song L, Wang X, Hu Y. Multi-functional hydroxyapatite/polyvinyl alcohol composite aerogels with self-cleaning, superior fire resistance and low thermal conductivity. *Compos Sci Technol* **158**, 128-136 (2018).

30. Gupta P, Singh B, Agrawal AK, Maji PK. Low density and high strength nanofibrillated cellulose aerogel for thermal insulation application. *Mater Des* **158**, 224-236 (2018).

31. Farooq M, Sipponen MH, Seppälä A, Österberg M. Eco-friendly flame-retardant cellulose nanofibril aerogels by incorporating sodium bicarbonate. *ACS Appl Mater Interfaces* **10**, 27407-27415 (2018).

32. Zhang X, Zhao X, Xue T, Yang F, Fan W, Liu T. Bidirectional anisotropic polyimide/bacterial cellulose aerogels by freeze-drying for super-thermal insulation. *Chem Eng J* **385**, 123963 (2020).

33. Seantier B, Bendahou D, Bendahou A, Grohens Y, Kaddami H. Multi-scale cellulose based new bio-aerogel composites with thermal super-insulating and tunable mechanical properties. *Carbohydr Polym* **138**, 335-348 (2016).

34. Song M, Jiang J, Qin H, Ren X, Jiang F. Flexible and super thermal insulating cellulose nanofibril/emulsion composite aerogel with quasi-closed pores. *ACS Appl Mater Interfaces* **12**, 45363-45372 (2020).

35. Qi J, *et al.* Lightweight, flexible, thermally-stable, and thermally-insulating aerogels derived from cotton nanofibrillated cellulose. *ACS Sustain Chem Eng* **7**, 9202-9210 (2019).

36. Zhao S, *et al.* Multiscale assembly of superinsulating silica aerogels within silylated nanocellulosic scaffolds: improved mechanical properties promoted by nanoscale chemical compatibilization. *Adv Funct Mater* **25**, 2326-2334 (2015).

37. Zhang X, Cheng X, Si Y, Yu J, Ding B. All-Ceramic and Elastic Aerogels with Nanofibrous-Granular Binary Synergistic Structure for Thermal Superinsulation. *ACS Nano* **16**, 5487-5495 (2022).

38. Yang J, *et al.* Superinsulating BNNS/PVA Composite Aerogels with High Solar Reflectance

for Energy-Efficient Buildings. *Nano-Micro Lett* **14**, 1-16 (2022).

39. Guo J, *et al.* Hypocrystalline ceramic aerogels for thermal insulation at extreme conditions. *Nature* **606**, 909-916 (2022).

40. Leroy A, *et al.* High-performance subambient radiative cooling enabled by optically selective and thermally insulating polyethylene aerogel. *Sci Adv* **5**, eaat9480 (2019).

41. Yang M, *et al.* Bioinspired "skin" with cooperative thermo-optical effect for daytime radiative cooling. *ACS Appl Mater Interfaces* **12**, 25286-25293 (2020).

42. Yue X, Wu H, Zhang T, Yang D, Qiu F. Superhydrophobic waste paper-based aerogel as a thermal insulating cooler for building. *Energy* **245**, 123287 (2022).

43. Oh MJ, Lee JH, Yoo PJ. Graphene‐Based Ultralight Compartmentalized Isotropic Foams with an Extremely Low Thermal Conductivity of 5.75 mW m− 1 K− 1. *Adv Funct Mater* **31**, 2007392 (2021).

44. Li T, *et al.* Anisotropic, lightweight, strong, and super thermally insulating nanowood with naturally aligned nanocellulose. *Sci Adv* **4**, eaar3724 (2018).

45. Shan X, *et al.* Aerogel‐Functionalized Thermoplastic Polyurethane as Waterproof, Breathable Freestanding Films and Coatings for Passive Daytime Radiative Cooling. *Adv Sci*, 2201190 (2022).

46. Zhou K, *et al.* Three-dimensional printable nanoporous polymer matrix composites for daytime radiative cooling. *Nano Lett* **21**, 1493-1499 (2021).

47. Mandal J, *et al.* Hierarchically porous polymer coatings for highly efficient passive daytime radiative cooling. *Science* **362**, 315-319 (2018).

48. Li X, Peoples J, Yao P, Ruan X. Ultrawhite BaSO4 paints and films for remarkable daytime subambient radiative cooling. *ACS Appl Mater Interfaces* **13**, 21733-21739 (2021).

49. An L, *et al.* Reflective paint consisting of mesoporous silica aerogel and titania nanoparticles for thermal management. *ACS Appl Nano Mater* **4**, 6357-6363 (2021).

50. Zhong H, Zhang P, Li Y, Yang X, Zhao Y, Wang Z. Highly solar-reflective structures for daytime radiative cooling under high humidity. *ACS Appl Mater Interfaces* **12**, 51409-51417 (2020).

51. Wang T, Wu Y, Shi L, Hu X, Chen M, Wu L. A structural polymer for highly efficient all-day

passive radiative cooling. *Nat Commun* **12**, 1-11 (2021).

52. Kim H, McSherry S, Brown B, Lenert A. Selectively enhancing solar scattering for direct radiative cooling through control of polymer nanofiber morphology. *ACS Appl Mater Interfaces* **12**, 43553-43559 (2020).

53. Zhang J, *et al.* Mechanically robust and spectrally selective convection shield for daytime subambient radiative cooling. *ACS Appl Mater Interfaces* **13**, 14132-14140 (2021).

54. Wang H-D, *et al.* Superhydrophobic porous film for daytime radiative cooling. *Appl Mater Today* **24**, 101100 (2021).

55. Wang T, Zhang Y, Chen M, Gu M, Wu L. Scalable and waterborne titanium-dioxide-free thermochromic coatings for self-adaptive passive radiative cooling and heating. *Cell Rep Phys Sci* **3**, 100782 (2022).

56. Tian Y, *et al.* Superhydrophobic and recyclable cellulose-fiber-based composites for high-efficiency passive radiative cooling. *ACS Appl Mater Interfaces* **13**, 22521-22530 (2021).

57. Xue X, *et al.* Creating an eco‐friendly building coating with smart subambient radiative cooling. *Adv Mater* **32**, 1906751 (2020).

58. Lin Y, Williams TV, Xu T-B, Cao W, Elsayed-Ali HE, Connell JW. Aqueous dispersions of few-layered and monolayered hexagonal boron nitride nanosheets from sonication-assisted hydrolysis: critical role of water. *J Phys Chem C* **115**, 2679-2685 (2011).

59. Zhang B, *et al.* High-efficient liquid exfoliation of boron nitride nanosheets using aqueous solution of alkanolamine. *Nanoscale Res Lett* **12**, 1-7 (2017).

60. Wang W, Chen SJ, De Souza FB, Wu B, Duan WH. Exfoliation and dispersion of boron nitride nanosheets to enhance ordinary Portland cement paste. *Nanoscale* **10**, 1004-1014 (2018).

61. Shi Y, *et al.* Synthesis of few-layer hexagonal boron nitride thin film by chemical vapor deposition. *Nano Lett* **10**, 4134-4139 (2010).

62. Lee D, Lee B, Park KH, Ryu HJ, Jeon S, Hong SH. Scalable exfoliation process for highly soluble boron nitride nanoplatelets by hydroxide-assisted ball milling. *Nano Lett* **15**, 1238-1244 (2015).
