# Supplementary material for: Scalable anisotropic cooling aerogels by additive freeze-casting
Source: Nat Commun. 2022 Sep 22;13:5553. doi: 10.1038/s41467-022-33234-8 (PMC9499976; doi:10.1038/s41467-022-33234-8)
Supplement: Supplementary file 3 — Description of Additional Supplementary Files [file 41467_2022_33234_MOESM3_ESM.pdf]

**Description of Additional Supplementary Files**

File Name: Supplementary Movie 1
Description: Additive freeze-casting process

File Name: Supplementary Movie 2
Description: Mechanical flexibility of large-scale ACA panel

File Name: Supplementary Movie 3
Description: Water resistance of ACA panel

File Name: Supplementary Movie 4
Description: Water resistance of PU coated ACA panel
